# Relationships between printability and rheology of inks for personalized nutrition

**DOI:** 10.1016/j.crfs.2025.101220

**Published:** 2025-10-15

**Authors:** Ruud van der Sman, Bei Tian, Seyed-Ali Ghoreishy, Martijn Noort

**Affiliations:** a Wageningen Food & Biobased Research, Wageningen University & Research, The Netherlands; b Food Process Engineering, Wageningen University & Research, The Netherlands

**Keywords:** Rheology, 3D printing, Printability, Image analysis, Personalized nutrition

## Abstract

This study explores the relationship between printability and rheology of 3D food printing inks developed for personalized nutrition. We have developed a wide variety of inks differing in macronutrient composition, with formulations either carbohydrate-rich, protein-rich, or fiber-rich. We have experimentally characterized their rheology and printability. Rheological measurements, obtained via strain sweeps, were analyzed by a descriptive model capturing the elastic modulus, tan(δ), the yield stress, the critical strain, and the strain-thinning index. Printability was assessed through the required printing force, and the printing accuracy via top-view image analysis.

The yield stress showed a strong correlation with the printing force. Moderate correlations were found between printing accuracy yield stress, tan(δ), and strain-thinning exponent. We attribute the moderate correlations to inhomogeneity of the inks imparted by their imperfect mixing and hydration. These issues merit further investigation in the context of 3D food printing.

However, interestingly, the yield stress correlated with the strain-thinning exponent, suggesting that higher yield stress inks require stronger strain/shear thinning to be printable. The correlations of printing accuracy with tan(δ), and strain-thinning exponents align with findings from previous studies. Together with results of earlier studies, a printability window could be constructed, holding for a wide range of food ink formulations. This is an important step towards developing inks for personalized nutrition.

## Highlights

• Printing Accuracy is evaluated with Image Analysis.
• Printing Force is strongly correlated with yield stress.
• Printing Accuracy is correlated with yield stress, tan(delta), and strain thinning.
• Yield stress of inks is correlated with tan(delta) and strain thinning.
• Printability Window as function of tan(delta), agrees with literature.

## 1 Introduction

In recent years, interest in 3D food printing for personalized nutrition has grown (Caulier et al., 2020, Venkatachalam et al., 2023, van der Sman et al., 2024, Bugday et al., 2024, Bugday et al., 2025). 3D-printed foods are easily customized in composition, texture, and shape, making them well suited to meet the nutritional requirements of specific consumer groups such as the elderly, dysphagia patients (Ekonomou et al., 2024), hospital patients, athletes, and military personnel (Caulier et al., 2020). However, 3D food printing inks are quite constrained in their rheological properties to ensure printability, typically requiring the ink to fall within a specific rheological window (Gholamipour-Shirazi et al., 2019, Maldonado-Rosas et al., 2022, García Tuñón et al., 2023). In this study, we examine the relationship between rheology and printability for several classes of food inks designed for personalized nutrition. Thereby, we cover a much wider range of food formulations than above mentioned earlies studies, investigating relation between rheology and printability.

We focus specifically on inks that are extruded at room temperature and stabilized by a yield stress. The design of printable inks with a prescribed macronutrient profile is an example of the general problem of designing yield stress fluids, which remains a complex and active area of research (Nelson and Ewoldt, 2017, Nelson et al., 2019). Ideally, we like to decouple nutrition value from rheological properties and thus printability. In a parallel project we have shown that there is potential with food inks based on pre-gelatinized starch as stabilizing hydrocolloid, with pea proteins, pea starch, and pea fibers as fillers (Bugday et al., 2024). However, these inks have nutritional value and textural properties far from ideal. In the current research project (van der Sman et al., 2024), we have strived for inks with attractive nutritional profile and textural properties. In this study we did not focus on optimizing nutritional value, but we focus on the relation between rheology and printability, for a wide variety of inks.

For extrusion-based food printing, key rheological properties include yield stress, shear-thinning, thixotropy, and elastic modulus (Gholamipour-Shirazi et al., 2019, Maldonado-Rosas et al., 2022, García Tuñón et al., 2023). Two main criteria determine printability: (1) the ink must flow easily through the narrow printer nozzle, and (2) it must solidify quickly after deposition to support subsequent layers. In order to flow through the nozzle, the food ink should exhibit a moderate yield stress, and strong shear-thinning behavior — reducing viscosity at the high shear rates encountered during extrusion. Extrusion printers are often limited in their maximal force, and if this force cannot overcome the yield stress $\sigma_{Y}$, the ink will not be extruded. Moreover, practical applications demand a minimum throughput, which translates into a minimum printing speed/shear rate ${\overset{˙}{\gamma }}_{min}$. As such, the maximum available force $F_{max}$ must exceed the stress at this minimum shear rate.

Assuming the food ink follows the Herschel–Bulkley model and flows through a straight cylindrical nozzle of radius R and length L, this condition can be expressed as:

(1) $$F_{max}=\Delta p\pi R^{2}=\sigma_{wall}2\pi RL=\sigma_{Y}\left(1+{\left(\frac{{\overset{˙}{\gamma }}_{min}}{{\overset{˙}{\gamma }}_{cr}}\right)}^{n}\right)2\pi RL$$

n is the so-called shear-thinning index, δp the pressure drop over the nozzle, $\sigma_{wall}$ is the wall shear stress. For strong shear thinning inks n<0.25. ${\overset{˙}{\gamma }}_{cr}$ is the critical shear rate, indicating the start of the shear-thinning regime.

To ensure self-support of the printed object, the yield stress must exceed the hydrostatic pressure due to gravity: $\sigma_{Y}>\sqrt{3}\rho gH$, where ρ is the mass density of the ink, g the gravity acceleration, and H the object height (M’barki et al., 2017, Perrot et al., 2021). Many food inks are thixotropic, meaning their yield stress decreases under high shear during extrusion and recovers over time post-deposition as the microstructure rebuilds — typically driven by thermal fluctuations. This time-dependent thixotropic behavior is quantified by the recovery time. If the recovery is too long, deposited layers may deform under the weight of the new layers. Thixotropy is commonly measured via 3ITT recovery tests (Heckl et al., 2023).

Interestingly, some studies have reported correlations between thixotropic recovery and viscoelastic properties, as characterized by the loss factor tan(δ) (Gholamipour-Shirazi et al., 2019, Heckl et al., 2023, Jeon et al., 2024, Aina et al., 2024), although these properties are typically considered independent (Ewoldt and McKinley, 2017, Wang and Ewoldt, 2023).

For our framework of 3D food printing for personalized nutrition, we have developed 3 classes of inks, either rich in starch, proteins, or food fibers (Venkatachalam et al., 2023, van der Sman et al., 2024, Bugday et al., 2024), previously characterized via amplitude sweeps (van der Sman et al., 2024). In this study, we investigate whether their rheology correlate with printability, which is assessed via the measured extrusion, and the printing accuracy, as determined via image analysis.

We perform a regression analysis to evaluate the relationship between printability (extrusion force and printing accuracy) and the inks’ rheological properties, using the model parameters reported in our earlier work (van der Sman et al., 2024). Finally, we interpret our findings in the context of existing literature.

## 2 Materials and methods

### 2.1 Materials

We have created three classes of food inks, either rich in carbohydrates, proteins, or fibers. The carbohydrate-rich inks are based on a cookie-dough formulation and included wheat flour, wheat bran, egg white powder, shortening, cold-swelling starch, leavening agents, salt, and a variety of plasticizers such as maltodextrin, sucrose, fructo-oligosaccharide, xylitol, and water. The fiber-rich inks were formulated using carrot fiber, pectin, citric acid buffer, and plasticizers like sucrose, soluble corn fiber, fructo-oligosaccharide, xylitol, and water. Protein-rich inks, inspired by protein bar recipes, contained calcium-caseinate, whey protein, κ-carrageenan, sunflower oil, and plasticizers like sucrose, glucose syrup, glycerol, soluble corn fiber, and water. Detailed formulations of each ink were already provided in our previous paper (van der Sman et al., 2024), but it is repeated in the Supplementary Materials. In total, we had 18 carbohydrate-rich doughs, 10 protein-rich, and 10 fiber-rich inks.

### 2.2 Mixing procedures

*Carbohydrate-rich inks*

The inks are prepared using a planetary mixer (Hobart Nederland B.V.) equipped with a stainless steel flat beater. The shortening is first conditioned to room temperature, then mixed with sugar, sodium bicarbonate, sodium acid pyrophosphate, salt, and egg white powder for 1 min at speed 1. Water is gradually added while mixing at speed 2 for a total of 4 min, ensuring complete hydration. Wheat flour and cold-swelling starch are then added and mixed at speed 1 until a homogeneous dough is formed. Finally, the mixture was briefly mixed for 10 s at speed 2 to ensure uniformity.

*Protein-rich inks*

The protein-rich inks are prepared using a Stephan cutter (UMC5), with the mixing bowl preheated at 65 °C. Whey protein isolate, calcium caseinate powder, and sunflower oil are mixed under 80% vacuum for 30 s. The vacuum is released, and glycerol, glucose-fructose syrup, and κ-carrageenan solution are added. Mixing continued under 80% vacuum for an additional 3 min at low speed. The κ-carrageenan solution is prepared separately at 80 °C and maintained at 65 °C before use. In formulations containing soluble corn fibre, it is added to the κ-carrageenan solution during its preparation.

*Fibre-rich inks*

The pectin and sugar are solubilized in water in a bath of 98 °C for 1.5 h. The resulting solution was transferred to a Hobart mixer and combined with a 50% citric acid solution, tri-sodium-citrate, and carrot powder. The paste is mixed at speed 1 for 2 min. After preparation, all the inks are stored overnight in a refrigerator before use.

### 2.3 3D printing design

The designed print shape is an object of 4 connected cylinders, printed on a flat bottom layer. The object should have 4-fold rotation symmetry, and mirror symmetry in the horizontal and vertical axes. The bottom layer dimensions are 40 × 40 mm, and the height is 15 mm.

Objects are printed using a TNO in-house manufactured printer, consisting of a syringe-based extruder with a stepper motor, 30 mL plastic syringe, temperature-controllable syringe holder and plunger, completed with a sensor measuring force up to 1200 N, with more details found in Ghorbani et al. (2025). For printing we have used a nozzle size = 1 mm, track width = 2.2 mm, and printing speed = 900 mm/min. Moreover, we selected a layer height equal to 80% of nozzle diameter, ensuring a compression of the material to improve the adhesion of the layers, and to avoid problems with dripping or die swell (Gharraei et al., 2024). Our printing procedure has been adapted from Paolillo et al. (2021).

### 2.4 Rheology

Ideally, for full characterization of yield stress fluids one performs flow curves, where shear stress is measured as function of shear rate. In that case one can obtain all parameters present in the Herschel–Bulkley model. However, earlier investigations have shown that yield stress fluids used as 3D food inks are prone to edge fracture, while performing flow curves (Bugday et al., 2024). Hence, one has to revert to oscillatory rheology. Given the amount of ink formulations that had to be screened, we reverted to strain-sweeps. Via LAOS in combination with the Sequence of Physical Processes (Rogers, 2012) one could obtain Herschel–Bulkley relevant parameters, but the measurements and data analysis is much time consuming. Consequently, we have used strain sweeps for rheological finger printing, as has been performed for many other 3D food printing papers (Gholamipour-Shirazi et al., 2019, Nijdam et al., 2021, Maldonado-Rosas et al., 2022, Bugday et al., 2024, van der Sman et al., 2024).

Amplitude sweep measurements were performed using a Physica MCR 301 rheometer (Anton Paar GmbH, Stuttgart, Germany) in oscillatory mode, following the procedure described in van der Sman et al. (2022). A plate-plate geometry with serrated plates (25 mm diameter) was used, with a fixed gap of 2 mm. Measurements are conducted at 25 °C. Samples are equilibrated for 5 min prior to the strain sweep, which ranged from 0.001% to 100% strain in a logarithmic step at a constant frequency of 1 Hz.

### 2.5 Imaging

**Fig. 1:**
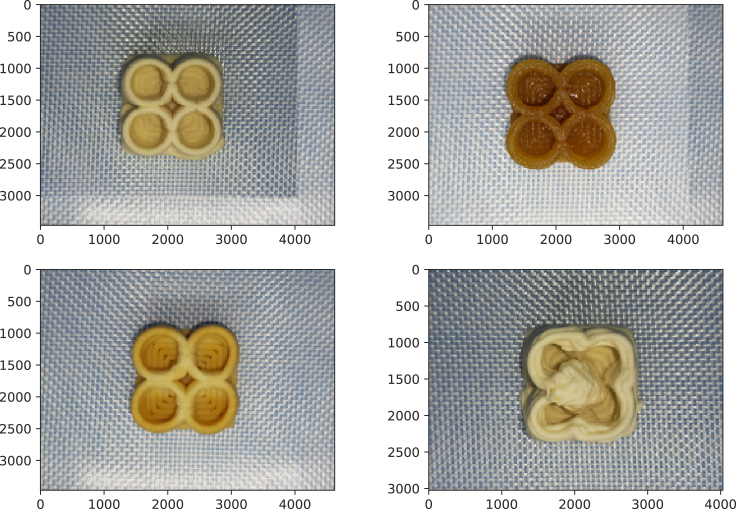
Top view images of printed objects using different inks: (a) protein-rich ink, (b) fiber-rich ink, (c) carbohydrate-rich (baked), and (d) collapsed object from protein-rich ink.

Top-view images of the samples are captured immediately after printing using a Samsung Galaxy A52 smartphone mounted at the top of the photo booth. To ensure consistent lightning and positioning, the samples are placed inside a BRESSER BR-PH50 Light Cube (50 × 40 × 39 cm), equipped with two fluorescent tubes for uniform, shadow-free illumination. Each sample is positioned 18.5 cm from the base using a fixed stand. The smartphone camera was aligned through an aperture at the top of the cube, at a height of 35 cm above the base, ensuring consistent distance, lightning and orientation for all images. Representative images are shown in Fig. 1.

### 2.6 Model description

A descriptive model is fitted to the strain sweep data for each ink. In previous studies, we have demonstrated that this model captures the rheological data of our 3D printing inks with a few parameters (van der Sman et al., 2024, Bugday et al., 2024). The model equations are:

$$G^{\prime }=G_{0}{\left[1+{\left(\frac{\gamma }{\gamma_{cr,1}}\right)}^{a}\right]}^{\frac{n_{1}-1}{a}}$$

(2) $$G^{\prime \prime }=\tan\left(\delta \right)G_{0}{\left[1+{\left(\frac{\gamma }{\gamma_{cr,2}}\right)}^{a}\right]}^{\frac{n_{2}-1}{a}}$$

The descriptive model has the following model parameters:

• $G_{0}$, the elastic modulus in the Linear Viscoelastic (LVE) regime,
• tan(δ), the ratio of $G^{\prime \prime }/G^{\prime }$ in the LVE regime
• the critical strains $\gamma_{cr,1}$ and $\gamma_{cr,2}$ signaling the end of the LVE, and the start of the strain-thinning regime.
• the strain-thinning (or strain-softening) indices $n_{1}$ and $n_{2}$

Materials fitting to this model are expected to exhibit yield stress behavior: if $\gamma <\gamma_{cr,1}$ the material behaves solid-like with $G^{\prime }\gg G^{\prime \prime }$, whereas for $\gamma \gg \gamma_{cr,1}$ it shows liquid-like (strain-thinning) behavior with $G^{\prime }\ll G^{\prime \prime }$. This softening is caused by the breakdown of the microstructure, which also explains the shear thinning behavior. The solid-to-liquid transition is defined by the yield stress, given by $\sigma_{Y}=G_{0}\gamma_{cr,1}$ (Dinkgreve et al., 2016). Strain thinning describes the decrease of $G^{\prime }$ with increasing strain, which happens beyond the critical strain $\gamma_{cr}$, and it is assumed due to breakdown of the equilibrium microstructure. Similar reasoning is held at the critical shear-rate during steady shear flow. Furthermore, for some materials there can be a relation between shear-thinning and strain-thinning index (Xu and Craig, 2011, Khandavalli and Rothstein, 2015), especially if $n_{1}/n_{2}=2$, one expects that $n=n_{2}$ (Miyazaki et al., 2006, van der Sman et al., 2023, van der Sman et al., 2024). But, we have found also exceptions to this rule amongst food inks (van der Sman et al., 2024). Furthermore, it is assumed that a=1.5 (van der Sman et al., 2024), and there is some correlation between (1) $\gamma_{cr,1}$ and $\gamma_{cr,2}$, and (2) $n_{1}$ and $n_{2}$ (Bugday et al., 2024).

## 3 Image analysis

**Fig. 2:**
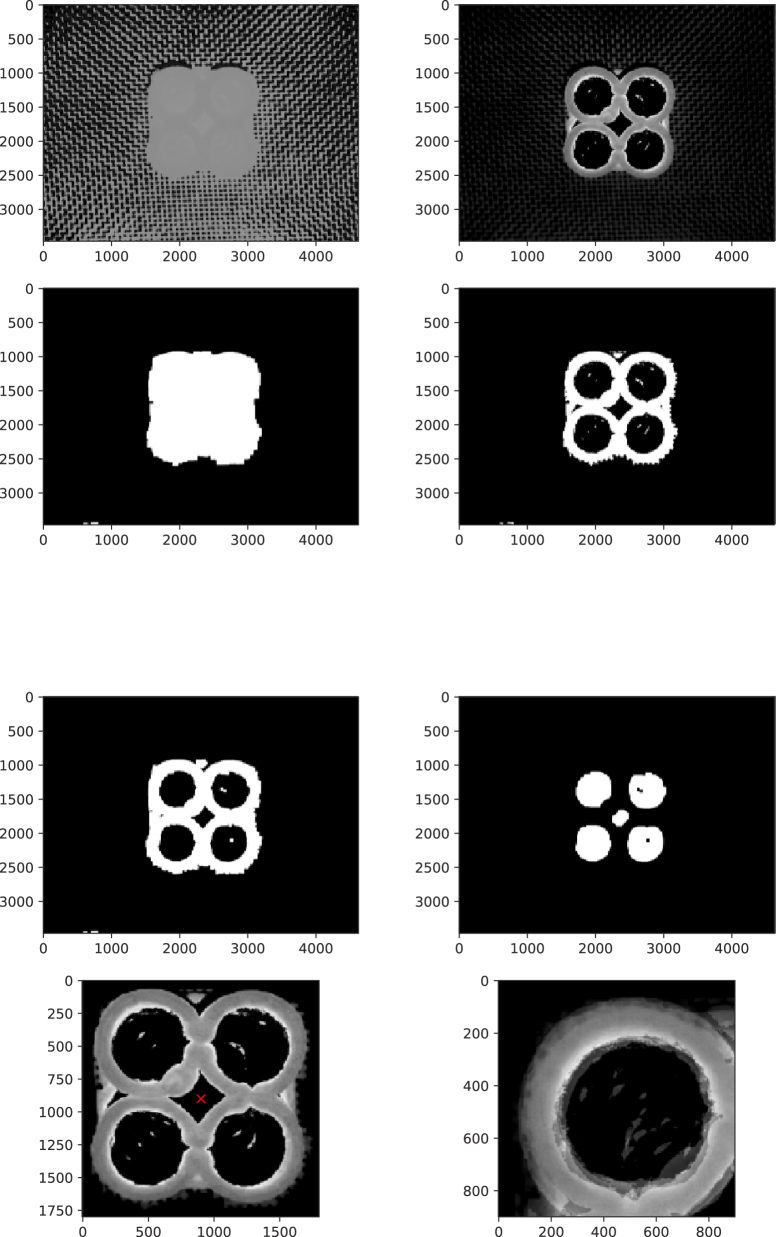
Steps in the image analysis algorithm. The top row shows *S-filter* and *C-filter*. The second row shows *S-mask* and *C-mask*. The third row shows *C-mask* after erosion/dilation, and inverted *C-mask* after flood-filling. The bottom row shows cropped *C-filter* with center of mass as a red cross and the averaged quarter image.

Image analysis is performed with Python using the OpenCV library. Each image underwent a pre-processing pipeline before quantitative characterization via the method of moments. The pre-processing steps are outlined below, with intermediate stages illustrated in Fig. 2.

*Algorithm*

1. Convert the image to the HSV ColorSpace
2. Filter the HSV channels for values within certain ranges, we created two filtered images *S-filter* and *C-filter*. The thresholds were different for each class of inks. For example, for dough inks, we used: *S-filter* = H *if* (80<H<120) OR (S>25), and *C-filter* = S *if* (H>90) AND (S<130) AND (V>100). The *S-filter* selects for the cylinders and the inner parts, while *C-filter* selects only the cylinder walls. Both *C-filter* and *S-filter* are binarized into *C-mask* and *S-mask*
3. Remove small blobs from the printing surface via erosion/ dilation from *S-mask* and *C-mask*
4. Flood fill *C-mask* background to remove any remaining blobs *C-mask* image is inverted, from which the centre of mass is determined.
5. Crop *C-filter* around the center of mass with image size $L_{x}=900$ pixels, using *S-mask* for masking The size of the mask is about the target size of the printed object.
6. Differences in illumination are corrected by dividing the grayscale of the cropped image by the average value
7. Cropped image is rotated over 90 degrees four times, and these four images are averaged. (For images with 4-fold symmetry, this operation should be invariant)
8. A quarter of the cropped image is taken, and is binarized using 1.25 the average value as a threshold

The binarized quarter image is analyzed using the method of methods (Teague, 1980, Teh and Chin, 1988) to extract key geometrical features: the centre of mass, the inner and outer radii. Pixel intensities in the binarized image are indicated by f(x,y). The zeroth order moment $M_{0,0}$ provides the total area (A) of the object:

(3) $$M_{0,0}=A=\underset{x}{\sum }\underset{y}{\sum }f\left(x,y\right)$$

The first-order moments yield the coordinates of the centre of mass:

$$C_{x}=M_{1,x}=\underset{x}{\sum }xf\left(x,y\right)/M_{0,0}$$

(4) $$C_{y}=M_{1,y}=\underset{y}{\sum }yf\left(x,y\right)/M_{0,0}$$

The second-order central moments, which are physically equivalent to the moment of inertia (I), are computed as:

$$M_{20}=\underset{x}{\sum }{\left(x-C_{x}\right)}^{2}f\left(x,y\right)$$

(5) $$M_{02}=\underset{y}{\sum }{\left(y-C_{y}\right)}^{2}f\left(x,y\right)$$

with the moment of inertia equal to $I=M_{20}+M_{02}$. Assuming the printed object approximates a cylindrical annulus, with outer radius $R_{2}$ and inner radius $R_{1}$, the area and moment of inertia are analytically expressed as:

(6) $$A=\pi \left(R_{2}^{2}-R_{1}^{2}\right)\text{;}I=\pi /4\left(R_{2}^{4}-R_{1}^{4}\right)$$

Solving this system of equations for the squared radii using Maple renders:

$$R_{1}^{2}=+\frac{A}{2\pi }+\frac{I}{A}$$

(7) $$R_{2}^{2}=-\frac{A}{2\pi }+\frac{I}{A}$$

**Fig. 3:**
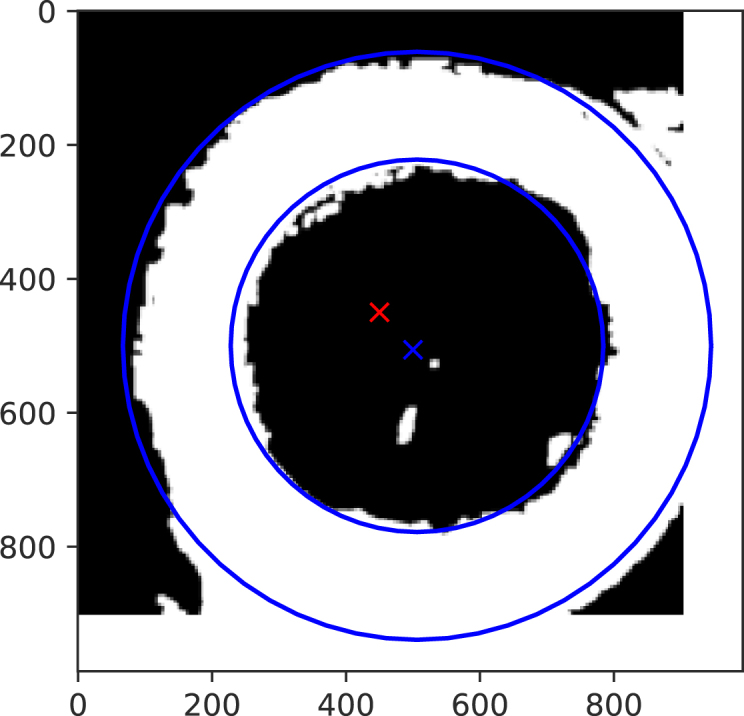
Final result of the image analysis algorithm, with centre of mass as blue cross, and estimated inner and outer radius indicated as blue circles obtained via the moments of the quarter image. The red cross shows the centre of the quarter image.

Fig. 3 illustrates the result of the moment analysis for a representative quarter image.

In a perfect printed object the cylinders touch their neighbors only at a single point, and the centre of mass would be located at $\left(C_{x},C_{y}\right)=\left(R_{2},R_{2}\right)$. However, in practice, deformation occurs due to track widening — caused by the weight of deposited layers, or the extrusion pressure imposed on the deposited layers. Consequently, this increases the thickness of the annulus thickness $R_{2}-R_{1}$. We quantify this deviation by comparing the measured ratio $R_{2}/R_{1}$, with its target value $R_{ratio}=1.34$ as given by the print design. Track widening also causes adjacent cylinders to adhere, shifting the center of mass away from its target position. $\left(\overset{¯}{C}/R_{2}-1\right)$, with $\overset{¯}{C}=\left(C_{x}+C_{y}\right)/2$, quantifies this deviation of the centre of mass of the cylinder from its target position. Anisotropic deformation such as wall collapse will distort symmetry in the image, leading to $C_{x}/C_{y}\neq 1$.

To capture all above mentioned deviations from the intended geometry, we define an empirical accuracy (dimensionless) metric Dev as:

(8) $$Dev^{2}={\left(R_{2}/R_{1}-R_{ratio}\right)}^{2}+{\left(C_{x}/C_{y}-1\right)}^{2}+{\left(\overset{¯}{C}/R_{2}-1\right)}^{2}$$

We applied equal weight factors in the definition of the metric Dev, as follows from the application of the entropic weight method (Dashore et al., 2013, Zhu et al., 2020). It shows that all three factors contribute about equally to the variation in Dev. The calculation procedure is briefly discussed in the Supplementary Material.

In Fig. 3 the blue cross indicates the actual position of the centre of mass $\left(C_{x},C_{y}\right)$, while the red cross indicates its target position. The two concentric blue circles show the radii $R_{1}$ and $R_{2}$. for the center of mass of the printed cylinder. Two effects of track widening are clearly visible: (1) misalignment between the red and blue crosses, and (2) an extended outer radius indicating inter-cylinder adhesion.

## 4 Results

### 4.1 Classification of inks based on rheology

The rheological behavior of the food inks is characterized through strain sweeps, as previously reported (van der Sman et al., 2024). The experimental data is analyzed with the descriptive model, Eq. (2). To visualize relationships between rheological parameters, we have constructed Ashby-style plots, color-coded by ink class: red for carbohydrate-rich, yellow for protein-rich, and green for fiber-rich inks. This classification is based on the dominant macronutrient component, as described in van der Sman et al. (2024).

First, the correlations between (1) $\gamma_{cr,1}$ and $\gamma_{cr,2}$, and (2) $n_{1}$ and $n_{2}$ are investigated, with results presented in figure A.1 The data confirms our hypothesis as indicated by the high correlation coefficient of $r^{2}>0.9$. Despite the strong global trends, the three ink classes exhibit distinctive clustering patterns in the Ashby plots. For the strain-thinning indices ($n_{1},n_{2}$), both carbohydrate-rich and protein-rich inks show a narrow range — typically $0.3<n_{2}<0.4$ — with few outliers exceeding $n_{2}=0.5$. In contrast, fiber-rich inks span a broader range of $n_{2}$ values and show weaker alignment with the global regression trend.

Regarding critical strains, carbohydrate-rich and protein-rich inks display a wide range of ($\gamma_{cr,1},\gamma_{cr,2}$) values that lie close to the regression line. Fiber-rich inks, however, exhibit less variation in $\gamma_{cr,1}$ and their data points tend to deviate more substantially from the trend line. In the following analysis it is assumed that $\gamma_{cr}=\gamma_{cr,1}=\gamma_{cr,2}$.

### 4.2 Correlation between printability and rheology

Printing accuracy was assessed through image analysis of the top views of printed objects. As a first step, we examined correlations between geometrical features derived from the method of moments. These correlations are shown in figure A.2 The ratio $C_{x}/C_{y}$ serves as a measure of asymmetry in the cylindrical shape. We observe that $C_{x}/C_{y}\approx 1$ for most samples, indicating minimal asymmetry. Accordingly, we define the mean center coordinate as $\overset{¯}{C}=\left(C_{x}+C_{y}\right)/2$.

The design value for the outer-to-inner radius ratio was $R_{ratio}=1.34$, indicated by the blue dashed line in the bottom-right panel. In nearly all formulations, we observe $R_{2}/R_{1}>1.34$, pointing to broadening of the printed track. This track width widening shows a moderate correlation with displacement of the center of mass, captured by $\overset{¯}{C}$. Among the ink classes, protein-rich inks appear more susceptible to track widening compared to carbohydrate- and fiber-rich inks.

In the next stage of analysis, we examined how the critical strain $\gamma_{cr}$ correlates with printing accuracy, quantified by the deviation metric Dev. As shown in the left panel of figure A.3, similar trends are observed across all ink classes, but with horizontal displacements between data sets.

To account for these shifts, we introduce a class-specific characteristic critical strain $\gamma_{char}$, and normalize the $\gamma_{cr}$ values accordingly. The right panel of figure A.3 shows the resulting collapse of the normalized data, revealing a unified trend across ink classes. The characteristic critical strains used for normalization were: $\gamma_{cr,char}=\left\{0.3,1.5,4\right\}\times 10^{-2}$ for doughs (red), protein-rich inks (yellow), and fiber-rich inks (green), respectively. The values were optimized via a least-squares minimization of the vertical distance between the rescaled data sets.

During the intermediate stage of analysis, we observe a similar dependency of $\gamma_{cr}$ with the printing accuracy Dev for the different ink classes. However, the data of each class was displaced along the horizontal axis by a certain amount, as shown in the left pane of figure A.3. Hence, we have determined for each ink class a characteristic $\gamma_{cr}$ (named $\gamma_{char}$) via which we will normalize the $\gamma_{cr}$ data, such that we obtained the optimal collapse of rescaled critical strain vs. printing accuracy, as shown in the right pane of figure A.3. The optimization of the collapse used a least-squares measure for the distance between data sets.

Next, we have analyzed the correlation between the printing accuracy Dev and rheology. Results of the regression analysis are shown in Fig. 5. Moderate correlations are observed with all rheological parameters: yield stress $\sigma_{Y}$, phase angle tan(δ), and the strain thinning index $n_{2}$. Similar to above, data points cluster by ink class. The correlation coefficients have improved slightly when excluding certain outliers from subclasses, as shown in figure A.4.

The printing force is directly measured during extrusion. Although the force tends to vary – particularly as the plunger nears the end of the syringe – we used only the maximum printing force for the correlation analysis. Fig. 6 presents these results.

A strong correlation is observed between printing force and yield stress $\sigma_{Y}$. Interestingly, protein-rich and carbohydrate-rich inks follow a common regression line, while fiber-rich inks align with a separate one. For each regression, the correlation coefficient exceeds $r^{2}>0.8$.

**Fig. 4:**
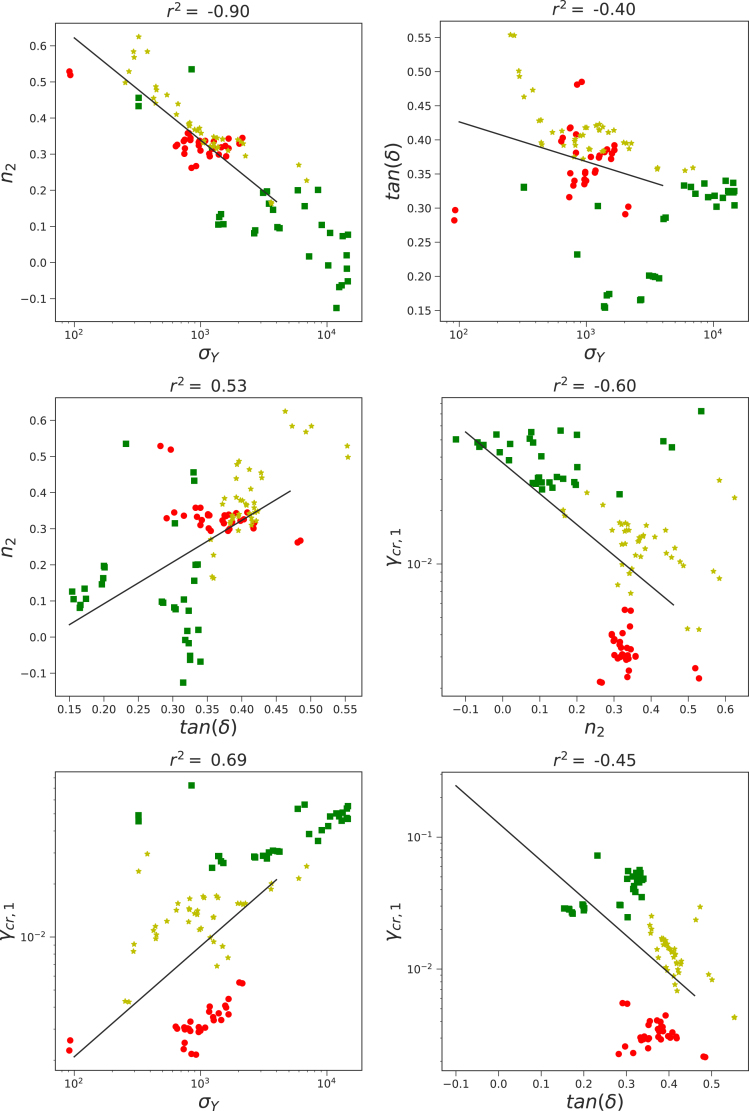
Correlations between rheological parameters, as obtained from fitting the amplitude sweeps. With colors, we have indicated the different ink classes: red = dough, yellow = protein-rich, green = fiber-rich. Correlation coefficients $r^{2}$ as determined with linear regression are stated in the figure titles.

Correlations between printing force and other rheological parameters are less pronounced. However, moderate correlations with tan(δ) suggest a relationship between viscoelasticity and extrusion behavior. Although no clear trend with the strain-thinning index $n_{2}$ is visible in Fig. 6, a moderate correlation between tan(δ) and $n_{2}$ was found in Fig. 4, consistent with previous studies (Gholamipour-Shirazi et al., 2019, Heckl et al., 2023, Jeon et al., 2024, Aina et al., 2024).

**Fig. 5:**
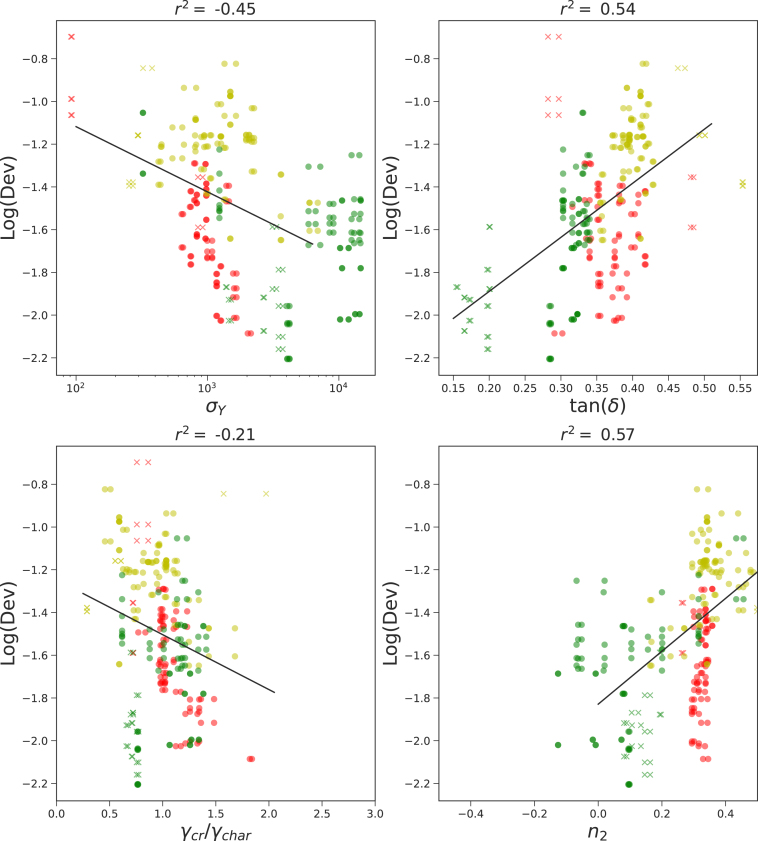
Correlations of rheological parameters with Dev. Correlation coefficients $r^{2}$ as determined with linear regression are stated in the figure titles.

**Fig. 6:**
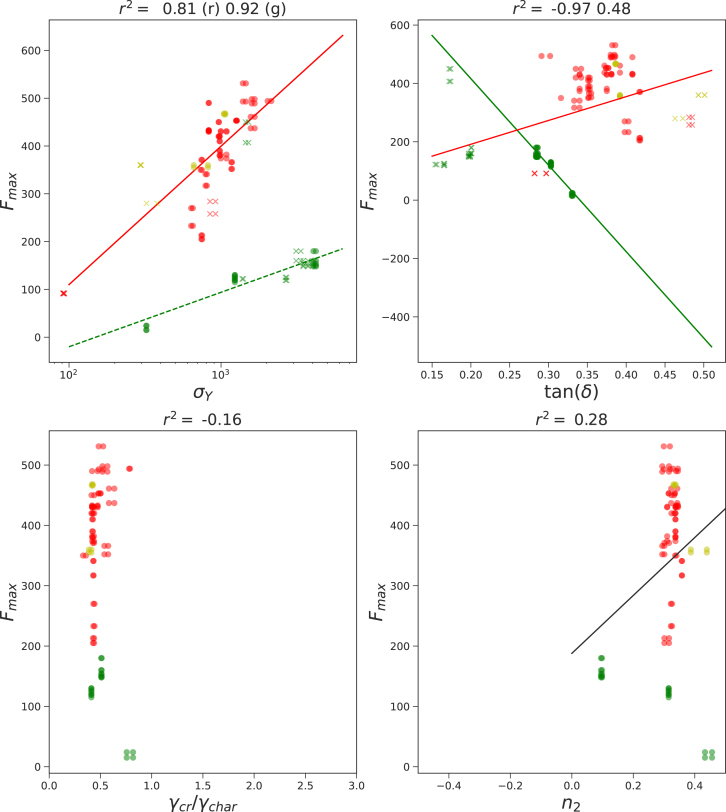
Printing force versus the rheological parameters for 3 classes of inks: carbohydrate-rich (red), protein-rich (yellow), and fiber-rich (green) inks. Subclasses are indicated with different shapes of symbols. Correlation coefficients $r^{2}$ as determined with linear regression are stated in the figure titles.

## 5 Discussion of results

Regarding the extrudability of food inks, we have found the strongest correlation between yield stress and printing force. This aligns with the Herschel–Bulkley equation, Eq. (1), which predicts a linear relation between the applied plunger force exerted and the yield stress. Within each ink class, the critical strain $\gamma_{cr}$ is relatively constant, suggesting a similar critical shear rate ${\overset{˙}{\gamma }}_{cr}$. Thus for $\overset{˙}{\gamma }>{\overset{˙}{\gamma }}_{cr}$ the printing force is expected to scale with $\sigma_{Y}$.

Interestingly, we have found a strong correlation between yield stress and strain thinning index. These parameters typically considered independent; however some studies reported a correlation between them for attractive gels (Nelson and Ewoldt, 2017). In contrast, for repulsive network fluids like microgel suspensions or emulsions, it is expected $n_{2}=0.5$, independent of yield stress (Erwin et al., 2010).

We hypothesize that this correlation arises from the rheological constraints imposed by the requirements of 3D printing. Inks with higher yield stresses must exhibit stronger shear thinning to remain printable within the force limitations of standard extrusion systems. In other words, printability may impose a functional dependency between $\sigma_{Y}$ and $n_{2}$.

As for printing accuracy, only moderate correlations are found with rheological parameters. Nevertheless, these observations are consistent with recent findings in 3D printing literature. We attribute the modest correlation strength to variations in ink properties imposed by their preparation, which requires proper hydration and mixing. The challenge of good mixing and hydration is shown by the study of Diañez et al. (2021). In this study they standardized the mixing with a continuous mixing device, but still observed significant fluctuations in rheology/viscosity, especially for inks with high viscosity and/or high amounts of dispersed/insoluble ingredients. Overall, it must be stated that mixing and hydration of complex food formulations using dry powder ingredients, remains underexplored in scientific literature. There is limited understanding of how to optimize the mixing of such systems (Mokhefi, 2024), especially those containing hydrocolloids — which tend to aggregate and form lumps (Wangler and Kohlus, 2018, Ong et al., 2020, Teichmann et al., 2024).

Further evidence of a link between viscoelasticity and flow behavior is provided by previous studies on other materials. For instance, in our earlier work on meat analogs (van der Sman et al., 2023), we also observed a correlation between the loss factor tan(δ) in the LVE regime and the strain-thinning/shear-thinning index $n_{2}$. If a material behaves as a critical gel (Winter, 1987), an exact analytical relation exists between tan(δ) and the power-law exponent m derived from frequency sweeps:

(9) tan(δ)=tan(mπ/2)

In such systems, both storage and loss moduli scale with angular frequency as $G^{\prime }∼G^{\prime \prime }∼\omega^{m}$. If the Cox–Merz or Rutgers–Delaware rule is assumed valid for these inks, then the exponent m can be related to the shear-thinning or strain-thinning index $n_{2}$.

**Fig. 7:**
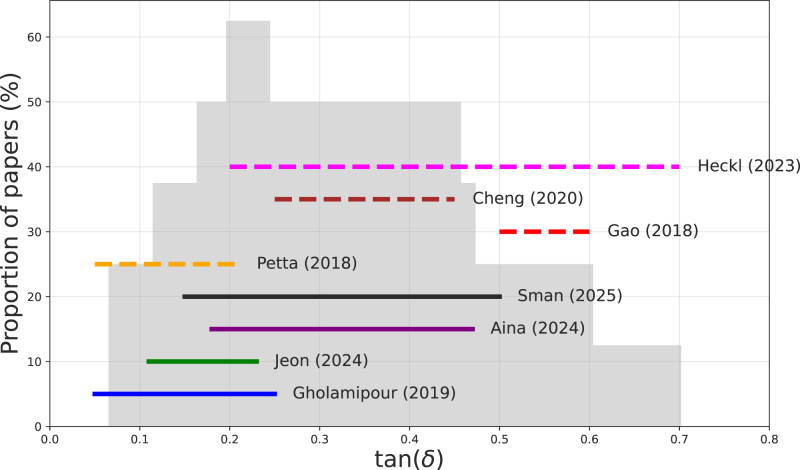
Rheological window for printability, based on multiple scientific papers on printability of food inks (solid lines), and bio-inks (dashed lines). The colored horizontal lines indicate the reported printability windows of papers, indicated by the reference at the right side of the line. The gray bar graph indicates the relative amount of papers reporting printability for the particular value of tan(δ), as indicated on the horizontal axis.

Several studies have formulated a “rheological window” for printability using tan(δ). These printability windows, together with our own window, are displayed in Fig. 7. Each window is indicated by a colored horizontal line, with the reference to the paper (with first author and publication year) printed next to the window. We have gathered data from both food inks as well as bio-inks, which are indicated by different linestyles. Regarding our printability window we have taken range of tan(δ) for which we have found multiple formulations with Dev<0.1. As one can observe in Fig. 5, our printability window is: 0.15<tan(δ)<0.5.

For several papers, which indicated critical gel behavior of the ink, we have translated their window into tan(δ) values using Eq. (9). Furthermore, it must be noted, that bio-inks have extra constraints regarding viability of the cell contained in the ink. To compare our results with the literature data, we have constructed a distribution, counting the relative number of papers having the particular value of tan(δ) in their printability window, which is shown as the gray bar graph in Fig. 7. Interestingly, our own results follow the median of this distribution. We view this as a good agreement of our results with these literature data.

A similar “rheological window” has been proposed for the adhesion behavior of adhesive polymer gels (Grillet et al., 2012). When tan(δ)<0.05, the material becomes overly elastic with a high yield stress. This impairs surface smoothness during deposition — since stress relaxation is too slow — and reduces interfacial adhesion.

The relevance of viscoelasticity for adhesion is further highlighted by the Dahlquist criterion, which states that good adhesive contact requires an elastic modulus $G_{0}$ below 100 kPa (Grillet et al., 2012). A stiffer material lacks sufficient viscous dissipation to deform and adhere effectively to a substrate.

On the other hand, materials that are too liquid-like (with short relaxation times and high tan(δ)) may also suffer from poor print fidelity. These inks can easily flow or creep, leading to deformation or detachment after deposition. Therefore, both upper and lower bounds on viscoelastic properties are essential for achieving optimal printing performance.

Adhesion plays a critical role in 3D printing, as deposited filaments must bond effectively with previously printed layers. Ideally, filaments should retain most of their designed diameter while deforming sufficiently to establish good interfacial contact with the underlying material.

Several studies have explored the link between thixotropy and adhesion in extrusion-based systems. For example, Lee et al. (2009) and Weng et al. (2021) investigated this relationship in the context of 3D-printed cement pastes and pharmaceutical gels. They propose that materials with short recovery times — i.e., those that rapidly rebuild their internal microstructure after extrusion — may lack sufficient time for molecular interdiffusion or bonding at the filament interface. This can limit interlayer adhesion. Conversely, materials with long recovery times may undergo more permanent plastic deformation during deposition. In such cases, the deformation required to achieve good contact becomes irreversible, enhancing mechanical interlocking and potentially improving adhesion. However, excessive plasticity can also compromise shape retention and dimensional accuracy. Thus, there exists a delicate balance: optimal print performance requires inks that deform just enough for good contact but recover quickly enough to maintain structural fidelity.

## 6 Conclusions

In this study, we have investigated the printability of a broad range of personalized nutrition inks by analyzing two metrics: the extrusion force, and the printing accuracy. Printing accuracy is quantified using image analysis of top views photographs of printed objects. Following object recognition, geometric features are extracted using the method of moments. Deviations of the printed object from the intended design are aggregated into a single number, Dev.

We have compared these printability indicators to rheology parameters of the inks, including the yield stress $\sigma_{Y}$, loss factor (tan(δ)), and strain thinning index ($n_{2}$). For the extrusion force, we obtained a strong correlation with the yield stress, consistent with the Herschel–Bulkley model. Notably, the yield stress itself was also correlated with $n_{2}$, even though these parameters are generally considered independent. We hypothesize that this correlation is imposed by the physical constraints of 3D printing — inks with high yield stress require more pronounced shear/strain thinning to be to remain extrudable at the set extrusion force.

As for printing accuracy, we have found moderate correlations of Dev with $\sigma_{Y}$, tan(δ), and $n_{2}$. We attribute the moderate strength of these correlations to heterogeneities introduced during the mixing and hydration of our complex, multi-component inks. The subject is proper mixing and hydration of complex food formulations is hardly investigated, with only some valuable exceptions. Likewise our study this literature indicates mixing and hydration should not be underestimated in importance, and it is particularly challenging if formulations include hydrocolloids and other water-soluble ingredients. The order-of-mixing and mixing temperature for each step must be carefully evaluated. In fact, this topic will be the subject of our follow-up project.

Nonetheless, our findings are consistent with those reported in literature. Several studies have noted correlations among tan(δ), strain/ shear thinning index, and thixotropic recovery. Similarly, our inks show moderate correlations between tan(δ) and the strain thinning index $n_{2}$, suggestion a possible interdependency of these rheological properties in food ink formulations.

For further research, we recommend to focus on (1) improving mixing and hydration procedures and (2) further exploring of the question whether correlations between rheological parameters are due to material properties, or a consequence of printability constraints.

## CRediT authorship contribution statement

**Ruud van der Sman:** Conceptualization, Initial draft, Software development, Writing – review & editing. **Bei Tian:** Measurement of rheology, Writing – review & editing. **Seyed-Ali Ghoreishy:** 3D printing, Measurement of printability. **Martijn Noort:** Project acquisition, Writing – review & editing.

## Declaration of competing interest

All authors declare that there are no conflicts of interest.

## Appendix A Supplementary data

MMC S1
