# Supplementary material for: Relationships between printability and rheology of inks for personalized nutrition
Source: Curr Res Food Sci. 2025 Oct 15;11:101220. doi: 10.1016/j.crfs.2025.101220 (PMC12615343; doi:10.1016/j.crfs.2025.101220)
Supplement: MMC S1 [file mmc1.pdf]

Suppplementary Materials to: Relationships between printability and rheology of inks for personalized nutrition

Ruud van der Sman[1,2], Bei Tian[1], Seyed-Ali Ghoreishy[1], Martijn Noort[1]

[1] *Wageningen Food & Biobased Research*

[2] *Food Process Engineering, Wageningen University*

Appendix A.

Appendix A.1. Additional figures

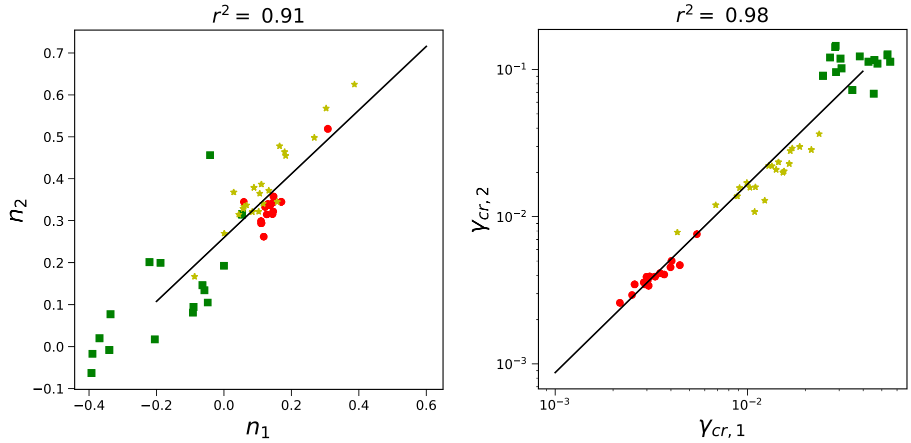

Figure A.1: Confirmation of expected correlations between rheological parameters regarding G' and G''. With colors, we have indicated the different ink classes: red=dough, yellow=protein-rich, green=fiber-rich. The black line indicates the result of the linear regression of all experimental data.

Email address: ruud.vandersman@wur.nl (Ruud van der Sman[1,2])

Appendix A.2. Entropy weight method

In the *Dev* metric we have three contributing factors: R_2/R_1 C_x/C_y, and $\bar{C}/R_2$, which are all dimensionless and of order unity. For each factor we have a series of N values for each measured sample. These experimental values are indicated with x_{ij}, with i indicating the sample, and j indicating one of the above three factors. The first step is to calculate the standardize values:

$$p_{ij} = \frac{x_{ij}}{\sum_i x_{ij}} \tag{A.1}$$

The entropy value of factor j is defined as:

$$E_j = -\frac{\sum_i p_{ij} \log(p_{ij})}{\log(N)} \tag{A.2}$$

The weight factor is calculated as:

$$w_j = \frac{1 - E_j}{\sum_j (1 - E_j)} \tag{A.3}$$

Appendix A.3. Formulations of the food inks

Table Appendix A.1: Formulation of 3DP cookie doughs

Ingredient	B1	B2	B3	B4	B5	B6	B7	B8	B9	B10	B11	B12	B13	B14	B15	B16	B17	B18
Flour WW	34.91	34.91	36.02	36.18	34.91	34.91	34.73	34.73	35.83	0.00	0.00	0.00	0.00	35.83	29.38	35.83	35.83	35.83
Flour ASW	0.00	0.00	0.00	0.00	0.00	0.00	0.00	0.00	0.00	35.83	0.00	0.00	0.00	0.00	0.00	0.00	0.00	0.00
Flour 1CW	0.00	0.00	0.00	0.00	0.00	0.00	0.00	0.00	0.00	0.00	35.83	0.00	0.00	0.00	0.00	0.00	0.00	0.00
Rice flour	0.00	0.00	0.00	0.00	0.00	0.00	0.00	0.00	0.00	0.00	0.00	35.83	0.00	0.00	0.00	0.00	0.00	0.00
Chickpea flour	0.00	0.00	0.00	0.00	0.00	0.00	0.00	0.00	0.00	0.00	0.00	0.00	35.83	0.00	0.00	0.00	0.00	0.00
Oatbran	0.00	0.00	0.00	0.00	0.00	0.00	0.00	0.00	0.00	0.00	0.00	0.00	0.00	0.00	6.45	0.00	0.00	0.00
WPI	4.11	4.11	4.24	4.26	4.11	0.00	4.09	0.00	0.00	0.00	0.00	0.00	0.00	0.00	0.00	0.00	0.00	0.00
Egg white	0.00	0.00	0.00	0.00	0.00	4.11	0.00	4.09	4.22	4.22	4.22	4.22	4.22	4.22	4.22	4.22	4.22	4.22
Starch	12.32	12.32	12.71	12.77	12.32	12.32	12.26	12.26	12.65	12.65	12.65	12.65	12.65	12.65	12.65	12.65	12.65	12.65
MDX DE6	3.08	0.00	0.00	0.00	0.00	0.00	0.00	0.00	0.00	0.00	0.00	0.00	0.00	0.00	0.00	0.00	0.00	0.00
MDX DE12	0.00	3.08	0.00	0.00	0.00	0.00	0.00	0.00	0.00	0.00	0.00	0.00	0.00	0.00	0.00	0.00	0.00	0.00
MDX DE18	0.00	0.00	0.00	0.00	3.08	3.08	3.06	3.06	0.00	0.00	0.00	0.00	0.00	0.00	0.00	0.00	0.00	0.00
Sucrose	11.29	11.29	11.65	11.70	11.29	11.29	11.24	11.24	11.59	11.59	11.59	11.59	11.59	11.59	11.59	11.59	5.80	0.00
FOS	0.00	0.00	0.00	0.00	0.00	0.00	0.00	0.00	0.00	0.00	0.00	0.00	0.00	0.00	0.00	0.00	2.90	5.80
Xylitol	0.00	0.00	0.00	0.00	0.00	0.00	0.00	0.00	0.00	0.00	0.00	0.00	0.00	0.00	0.00	0.00	2.90	5.80
Shortening	21.56	21.56	22.25	22.34	21.56	21.56	21.45	21.45	22.13	22.13	22.13	22.13	22.13	22.13	22.13	22.13	22.13	22.13
NaCl	0.41	0.41	0.42	0.43	0.41	0.41	0.41	0.41	0.42	0.42	0.42	0.42	0.42	0.42	0.42	0.42	0.42	0.42
Na2CO3	0.00	0.00	0.00	0.00	0.00	0.00	0.20	0.20	0.20	0.20	0.20	0.20	0.20	0.20	0.20	0.20	0.20	0.20
SAPP	0.00	0.00	0.00	0.00	0.00	0.00	0.30	0.30	0.31	0.31	0.31	0.31	0.31	0.31	0.31	0.31	0.31	0.31
Water	12.32	12.32	12.71	12.32	12.32	12.32	12.26	12.26	12.65	12.65	12.65	12.65	12.65	12.65	12.65	12.65	12.65	12.65

Table Appendix A.2: Formulation of 3DP cellular-based inks

Carrot Fiber Inks	C1	C2	C3	C4	C5	C6	C7	C8	C9	C10
Apple Puree	0	0	0	0	0	0	0	0	61.5	63.6
Carrot puree	0	0	0	0	0	85	85	85	0	0
Carrot Powder	15	10	90	85	80	14.2	13.8	13.4	0	0
kappa-carrageenan	0	0	0	0	0	0.8	1.2	1.6	0	0
Pectin HM	2	2	0	0	0	0	0	0	2	0.5
Water	33	35	10	15	20	0	0	0	0	0
Sugar	50	53	0	0	0	0	0	0	36.5	35.9
Color Code	g	g	y	y	y	k	k	k	r	r

Table Appendix A.3: Formulation of 3DP protein-bar inks

Ingredients	D1	D2	D3	D4	D5	D6	D7	D8	D9	D10
Whey Protein Isolate	15	15	15	15	15	15	15	15	15	15
Calcium Caseinate	25	25	25	25	25	25	25	25	25	25
Kappa-carrageenan	0.8	0.8	0.8	0.8	0.8	0.8	0.8	0.8	0.8	0.8
Water	24.2	24.2	27.6	27.6	25.9	25.9	24.2	24.2	24.2	24.2
Glycerol	7.5	7.5	7.5	7.5	7.5	7.5	0	0	0	0
Glucose Syrup	22.5	22.5	11.3	11.3	16.9	16.9	30	30	22.5	22.5
Sucrose	0	0	0	0	0	0	0	0	7.5	7.5
Soluble Corn Fiber	0	0	7.9	7.9	3.9	3.9	0	0	0	0

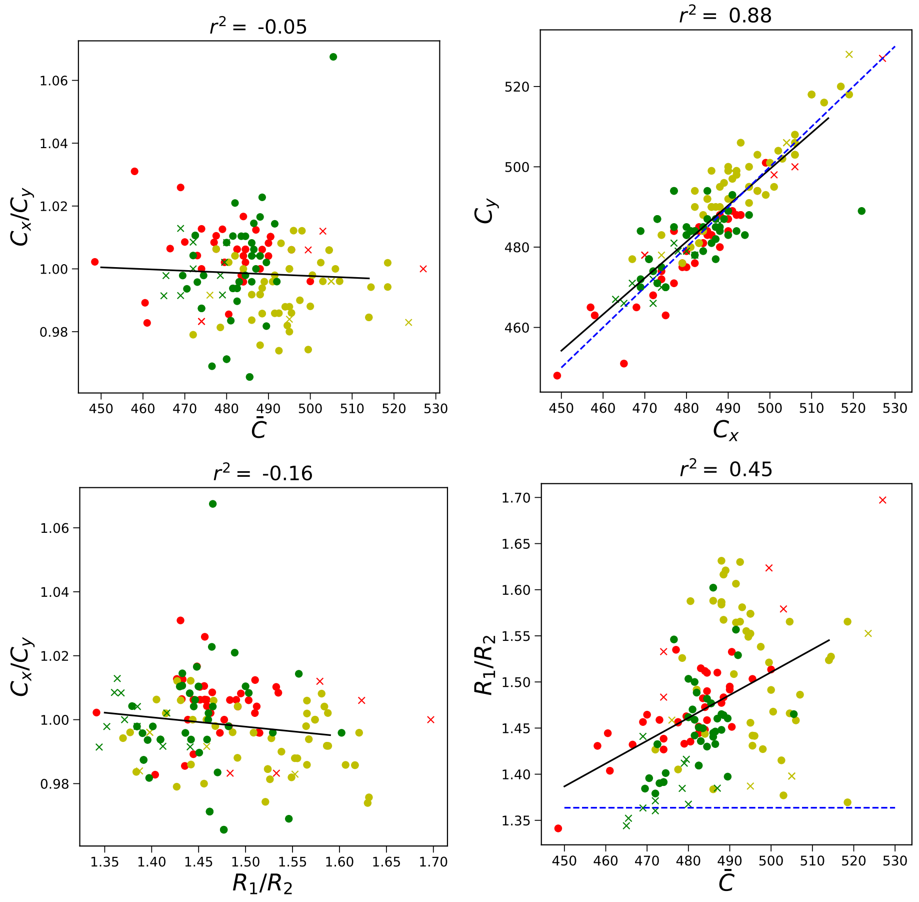

Figure A.2: Correlations between geometrical factors obtained from image analysis. Ink classes are color-coded: red = dough (carbohydrate-rich), yellow = protein-rich, green = fiber-rich. Blue dashed lines indicate a separate correlation for the fiber rich-inks.

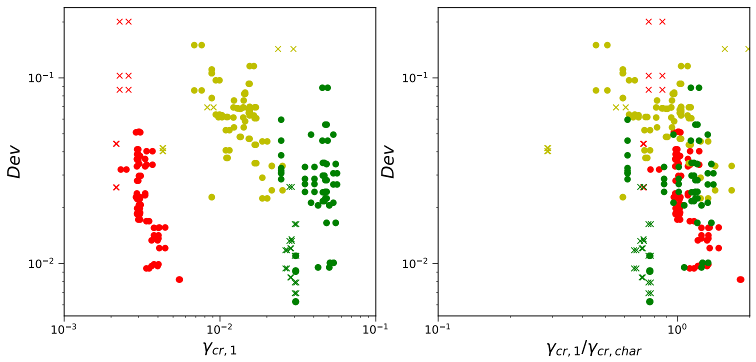

Figure A.3: Correlation between critical strain and printing accuracy (Dev). Left: unscaled data; Right: data rescaled by class-specific critical strain γ_{char}.

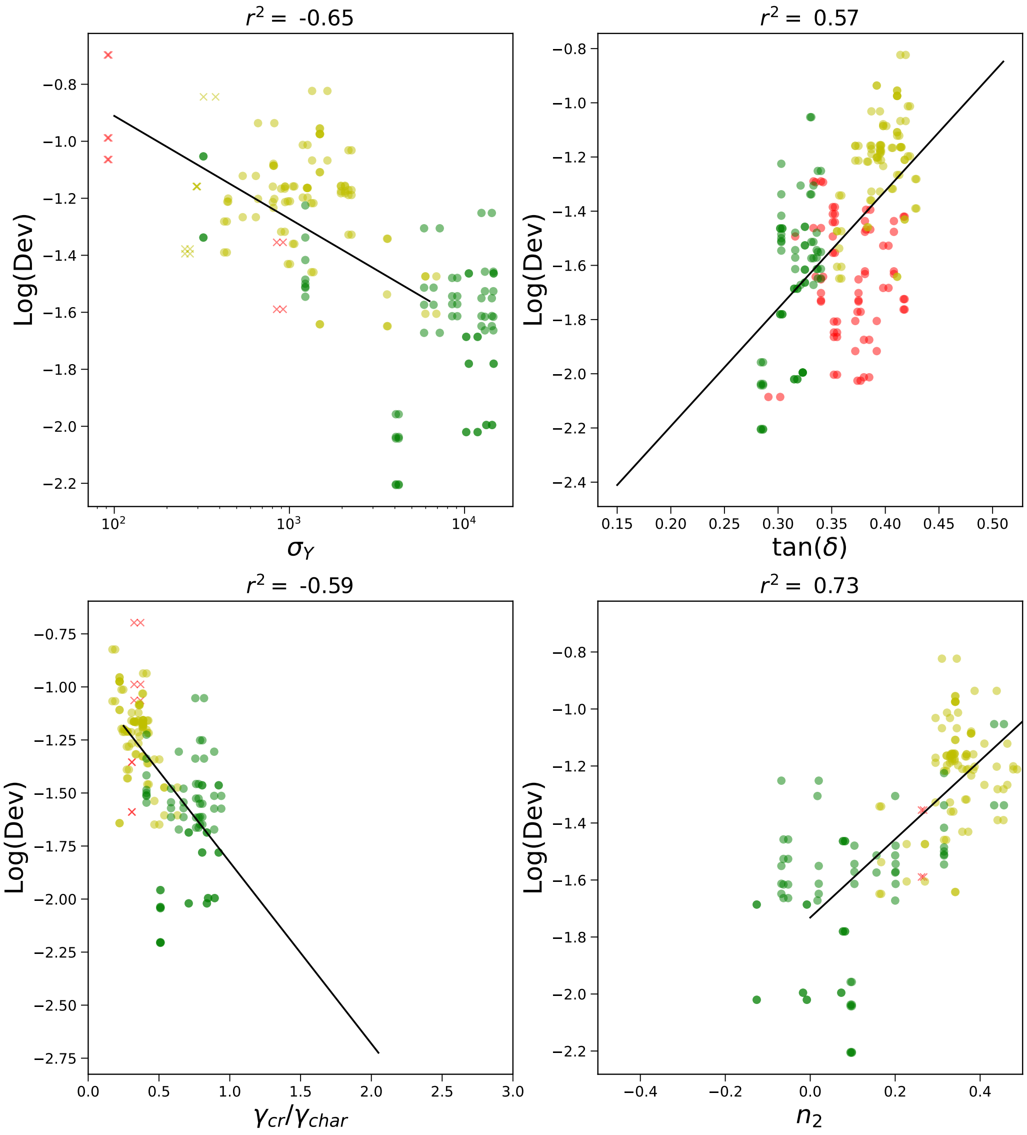

Figure A.4: Correlations of rheological parameters with *Dev*, with only several subclasses included (as indicated by their symbols).
